# Automation of ReactomeFIViz via CyREST API

**DOI:** 10.12688/f1000research.14776.2

**Published:** 2018-05-23

**Authors:** Fred Loney, Guanming Wu

**Affiliations:** 1 Knight Cancer Institute, Oregon Health & Science University, Portland, OR, USA; 2 Department of Medical Informatics and Clinical Epidemiology, Oregon Health & Science University, Portland, OR, USA

**Keywords:** ReactomeFIViz, CyREST, Cytoscape, Reactome, Pathway and network

## Abstract

Pathway- and network-based approaches project seemingly unrelated genes onto the context of pathways and networks, enhancing the analysis power that cannot be achieved via gene-based approaches. Pathway and network approaches are routinely applied in large-scale data analysis for cancer and other complicated diseases. ReactomeFIViz is a Cytoscape app, providing features for researchers to perform pathway- and network-based data analysis and visualization by leveraging manually curated Reactome pathways and highly reliable Reactome functional interaction network. To facilitate adoption of this app in bioinformatics software pipeline and workflow development, we develop a CyREST API for ReactomeFIViz by exposing some major features in the app. We describe a use case to demonstrate the use of this API in a Python-based notebook, and believe the new API will provide the community a convenient and powerful tool to perform pathway- and network-based data analysis and visualization using our app in an automatic way.

## Introduction

Pathway- and network-based computational approaches are now routinely used in large-scale data analysis to uncover hidden patterns that are otherwise impossible to discover. These approaches project significant genes, proteins, metabolites, and other kinds of biological entities collected from other approaches onto the context of pathways and networks, knowledge produced by many years’ experimental studies. Cytoscape ^1^ is the most popular biological network visualization and analysis platform, widely used in the research community to perform pathway and network analysis and visualization. The release of the CyREST app ^2^ enables Cytoscape as an integrative and indisposable tool to build automatic software pipeline and workflow in programming languages widely used by the bioinformatics and computational biology community, including Python and R, via a RESTful API. The standalone Java-based Cytoscape application thereby functions as a microservice servlet exposing the major features of Cytoscape.

Reactome ^3^ is the most comprehensive open source biological pathway knowledgebase, widely used in the research community, with its web site accessed by roughly 60,000 unique IP addresses per month. To perform genome-scale network-based data analysis and visualization, we have also constructed a highly reliable Reactome functional interaction (FI) network by extracting FIs from manually curated pathways from Reactome and other popular large-scale pathway databases and predicting FIs based on a machine learning approach ^4^. Based on this FI network and the high quality Reactome pathways, we have developed a Cytoscape app, called “ReactomeFIViz” ^5^, which is one of most popular Cytoscape apps, downloaded over 30,000 times since it was released in September, 2013 into Cytoscape app store.

ReactomeFIViz provides a suite of features to help users to perform pathway- and network-based data analysis and visualization for cancer and other complicated diseases. Users can construct a FI subnetwork based on a set of genes, perform network clustering, annotate found network modules using Reactome pathways and Gene Ontology terms, and perform survival analysis if clinical data is available to search for gene signatures related to patient overall survival. Users can also explore Reactome pathways directly inside Cytoscape, perform pathway enrichment analysis for a set of genes, and conduct pathway modeling using multiple types of omics data based on factor graphs converted automatically from Reactome pathways. Recently we added new features for users to visualize FDA approved cancer drugs and their targets interactions in the context of Reactome pathways and the FI network, and perform fuzzy logic based modeling to study the effects of drug application on the pathway activities (see ReactomeFIViz wiki page).

To facilitate third-party software tool developers to integrate the powerful network and pathway analysis features provided in ReactomeFIViz, we implemented a new CyREST API. The current version of this API is focused on FI network construction and Reactome pathway enrichment analysis for a set of genes.

## Methods

To develop the CyREST API for ReactomeFIViz, we followed the recommended procedures described in Cytoscape wiki on adding Automation to existing apps. To handle the complex data models used in ReactomeFIViz, we chose the Functions over Commands approach by adding JAX-RS resource onto existing ReactomeFIViz code base. In brief, we added two new Java packages, org.reactome.cytoscape.rest and org.reactome.cytoscape.rest.tasks, and refactored original tasks into ObservableTask. All refactored ObservableTasks are grouped into package org.reactome.cytoscape.rest.tasks, and their execution is managed by a SyncrhonousTaskManager object and monitored by their respective TaskObserver objects.

The ReactomeFIViz CyREST API is specified in a Java interface, ReactomeFIVizResource, and documented using Swagger UI as Java annotations for methods defined in the interface. The implementation of ReactomeFIVizResource is provided in class ReactomeFIVizResourceImp. Both the interface and the implementation are placed in package org.reactome.cytoscape.rest.

**Figure 1.**
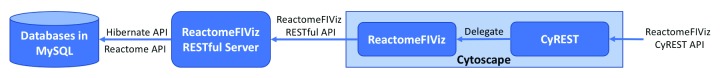
Software architecture for ReactomeFIViz CyREST API.

The ReactomeFIViz CyREST API is powered up by the CyREST app using its embedded light-weight Grizzly HTTP server. CyREST delegates all RESTful API calls to ReactomeFIViz, which then calls the ReactomeFIViz RESTful server via its RESTful API. The ReactomeFIViz server fetches the Reactome content from databases hosted in a MySQL database engine via a Hibernate API and the in-house built Reactome Java API ( Figure 1).

## Operation

Currently, the ReactomeFIViz CyREST API provides 8 methods ( Table 1). These methods allow third-party workflow and pipeline developers to construct a FI sub-network based on a set of genes listed in a variety of file formats, annotate displayed network using collected pathways, GO biological process, molecular function, or cellular component terms, perform network cluster and then annotate network modules. These methods also allow them to perform Reactome pathway enrichment analysis for a set of genes and then export pathway diagrams. The CyREST API document for ReactomeFIViz, which is accessed via menu Help/Automation/CyREST API, provides detailed description about all these resources.

**Table 1.** List of ReactomeFIViz CyREST API.

URL	HTTP Method	Function	Note
fiVersions	GET	List supported versions of the FI network	Usually three versions should be listed
buildFISubNetwork	POST	Build a FI subnetwork for a set of genes	Multiple file formats are supported
enrichment/{type}	GET	Perform network enrichment analysis	Four types are supported, which is specified by type in URL: Pathway, GO BP ^1^, MF ^2^, and CC ^3^
cluster	GET	Perform network clustering analysis	Clustering is performed for the displayed network
moduleEnrichment/{type}	GET	Perform network module enrichment analysis	Four types are supported, which is specified by type in URL: Pathway, GO BP, MF, and CC
pathwayTree	GET	Show the Reactome pathway tree	All pathways are returned from this resource
ReactomePathwayEnrichment	POST	Perform pathway enrichment analysis for a set of genes	pathwayTree must be called first
exportPathwayDiagram	POST	Export a pathway diagram as a PDF file	Only pathways with hasDiagram values equal to true can export diagrams.

Notes: 1. GO BP: Gene Ontology biological process; 2. MF: Molecular function; 3. CC: Cellular component.

## Results

ReactomeFIViz CyREST API provides a set of URL-based language-neutral functions, which accept parameters and return results in the JSON format. As with any other CyREST API, it can be easily integrated into Python, R, or any other programming language as long as it supports HTTP-based function calling. Here we describe a use case based on The Jupyter Notebook to showcase the usage of this API in a workflow development.

### Workflow

Previous study ^6^ has shown the genomic similarity between high-grade serous ovarian tumors and basal-like breast tumors based on multiple omics data types, including copy number variants (CNVs), somatic mutations, and mRNA gene expression. To demonstrate use of the ReactomeFIViz CyREST API, we perform a network- and pathway-based comparison analysis between genes having somatic mutations in TCGA ovarian cancer and breast cancer. Our analysis is focused on showing the utility of our API. Therefore, we use all TCGA breast cancer samples without subtyping to simplify our workflow.

**Figure 2.**
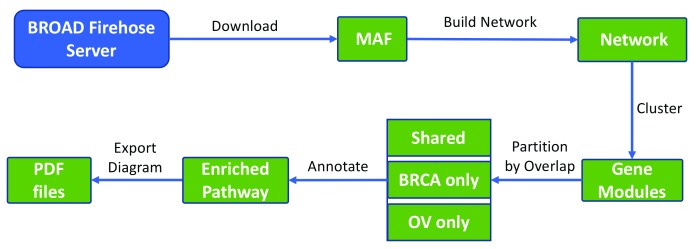
The workflow of the comparison analysis use case using TCGA OV and BRCA mutation data.

Figure 2 shows the workflow of this use case, and Table 2 lists the detailed information for actions performed in the workflow. The TCGA BRCA (breast invasive carcinoma) and OV (ovarian serous cystadenocarcinoma) mutation data was downloaded from the Broad firehose web site in the mutation annotation file (MAF) format using its RESTful API, and stored in two local files, one for each cancer type. The MAF file was then loaded into Cytoscape via the CyREST API, buildFISubNetwork, to construct a FI sub-network after choosing a sample cutoff to select genes forming a network composed of about 500 genes. The FI-network was then subject to network clustering analysis using the cluster call. Two sets of network modules from network clustering were compared to find modules shared and not shared between these two cancers in the Python notebook. Pathway enrichment analysis was performed using ReactomePathwayEnrichment to collect pathways not shared between them. These results suggest common and cancer-specific network and pathway patterns, facilitating researchers to understand shared and specific oncogenesis mechanisms in these two cancers. Finally, pathway diagrams were exported via exportPathwayDiagram.

**Table 2.** List of actions performed in the use case workflow.

Action	Workspace	URL	Sample Parameters
Download	Broad firehose web site	http://firebrowse.org/api/v1/Analyses/Mutation/MAF	format=‘tsv’ cohort=‘BRCA’
Build network	Cytoscape	http://localhost:1234/reactomefiviz/v1/buildFISubNetwork	file=‘BRCA.maf’ sampleCutoffValue=4
Cluster	Cytoscape	http://localhost:1234/reactomefiviz/v1/cluster	none
Partition by overlap	Python notebook	n/a	cutoff=.05
Annotate	Cytoscape	http://localhost:1234/reactomefiviz/v1/ReactomePathwayEnrichment	data=[‘ABCA1’, ‘ACL2’, ..., ‘ZNF462’]
Export diagram	Cytoscape	http://localhost:1234/reactomefiviz/v1/exportPathwayDiagram	dbId=68877 pathwayName=‘Mitotic Prometaphase’ fileName=‘Mitotic Prometaphase.pdf’

### Analysis results

We performed a network comparison study for the two FI subnetworks constructed separately for TCGA BRCA and OV mutated genes. Network clustering yielded 23 and 38 modules with the TCGA BRCA and OV FI subnetwork, respectively. Overlapping analysis for modules having no less than 3 genes showed 16 out of 18 BRCA modules significantly overlapped with 15 out of 22 OV modules (FDR < 0.05. Results not shown here. See the output in the notebook for details), implying that almost all of BRCA modules can be found in the OV subnetwork and some of BRCA modules are shared with more than one OV module. For example, BRCA Module 1 is significantly overlapped with OV Modules 2 and 3.

For the pathway comparison study, we focused on pathways enriched for network modules not shared between BRCA and OV, searching for possible different oncogenic mechanisms for these two cancers. The module overlapping analysis showed that BRCA modules 13 and 17, which contains 6 and 4 genes, respectively, are not significantly shared with any OV module using FDR cutoff = 0.05, though detailed analysis showed that Module 13 has one gene, ADCY9, shared with OV Module 1, and Module 17 has another gene, FLG, shared with OV Module 7. Pathway annotation for these two modules revealed that genes in there are significantly enriched for some signaling pathways, including DAG and IP3 signaling, Signaling by GPCR, and Glucagon signaling in metabolic regulation. There are 7 modules in the OV subnetwork that are not significantly shared with the BRCA subnetwork. Pathway enrichment analysis showed that genes in these modules are significantly involved in Ion channel transport, O-linked glycosylation, C-type lectin receptors, and several others. For detailed results, see the output from the notebook.

To visualize mutated genes in the context of Reactome pathways, the notebook also generated two pathway diagrams, one for each cancer, and saved into the working directory as PDF files. Entities in pathway diagrams composed of mutated genes are highlighted in purple.

## Discussion

Reactome provides a large set of high-quality manually curated pathways. The Reactome FI network provides a genome-scale highly reliable functional interaction network covering over 60% of total human genes. The ReactomeFIViz CyREST API delivers language neutral REST-based functions for third-party software developers to leverage high-valued resources provided by the Reactome project in their own software tools.

The current set of functions implemented in this version of ReactomeFIViz CyREST API focuses on some major features implemented in ReactomeFIVz, related to FI network construction, clustering, and Reactome pathway enrichment analysis. As shown in the above use case, it is very easy to integrate with other CyREST APIs and integrated into a Python or R programming language environment to perform Reactome-related pathway and network analysis and visualization.

We will expose other ReactomeFIViz features in the CyREST API, including gene expression data analysis, network module-based survival analysis, pathway modeling based on Boolean network and probabilistic graph model, and cancer drug visualization and simulation. We will also develop a Python package for easy third-party tool integration.

## Software availability

Home page for user guide: https://reactome.org/tools/reactome-fiviz

Cytoscape app store: http://apps.cytoscape.org/apps/reactomefiplugin

Latest source code: https://github.com/reactome-fi/CytoscapePlugIn/tree/path-x

Use case Jupyter notebook: https://github.com/reactome-fi/workflows

Source code at the time of publication: https://github.com/reactome-fi/CytoscapePlugIn/releases/tag/f1000_auto_paper for ReactomeFIViz, https://github.com/reactome-fi/workflows/releases/tag/f1000_auto_paper for the Python use case notebook

Archived source code at the time of publication: http://doi.org/10.5281/zenodo.1226433 ^7^ for ReactomeFIViz, http://doi.org/10.5281/zenodo.1226430 ^8^ for the Python use case notebook

License: The Creative Commons Attribution 3.0 Unported License ( http://www.reactome.org/?page_id=362).

## Data availability

The data referenced by this article are under copyright with the following copyright statement: Copyright: © 2018 Loney F and Wu G

Data associated with the article are available under the terms of the Creative Commons Zero "No rights reserved" data waiver (CC0 1.0 Public domain dedication). 

Output from the network comparison are available in the Python use case notebook http://doi.org/10.5281/zenodo.1226430 ^8^

## References

[1] Shannon P Markiel A Ozier O: Cytoscape: a software environment for integrated models of biomolecular interaction networks. *Genome Res.* 2003;13(11):2498–504. 10.1101/gr.1239303 14597658 PMC403769

[2] Ono K Muetze T Kolishovski G: CyREST: Turbocharging Cytoscape Access for External Tools via a RESTful API [version 1; referees: 2 approved]. *F1000Res.* 2015;4:478. 10.12688/f1000research.6767.1 26672762 PMC4670004

[3] Fabregat A Jupe S Matthews L: The Reactome Pathway Knowledgebase. *Nucleic Acids Res.* 2018;46(D1):D649–D655. 10.1093/nar/gkx1132 29145629 PMC5753187

[4] Wu G Feng X Stein L: A human functional protein interaction network and its application to cancer data analysis. *Genome Biol.* 2010;11(5):R53. 10.1186/gb-2010-11-5-r53 20482850 PMC2898064

[5] Wu G Dawson E Duong A: ReactomeFIViz: a Cytoscape app for pathway and network-based data analysis [version 2; referees: 2 approved]. *F1000Res.* 2014;3:146. 10.12688/f1000research.4431.2 25309732 PMC4184317

[6] Cancer Genome Atlas Network: Comprehensive molecular portraits of human breast tumours. *Nature.* 2012;490(7418):61–70. 10.1038/nature11412 23000897 PMC3465532

[7] Wu G: reactome-fi/CytoscapePlugIn: F1000Research ReactomeFIViz Automation (Version f1000_auto_paper). *Zenodo.* 2018 Data Source

[8] Loney F Wu G: reactome-fi/workflows: F1000Research ReactomeFIViz Automation (Version f1000_auto_paper). *Zenodo.* 2018 Data Source

