# Supplementary material for: Automation of ReactomeFIViz via CyREST API
Source: F1000Res. 2018 May 23;7:531. Originally published 2018 May 2. [Version 2] doi: 10.12688/f1000research.14776.2 (PMC5998009; doi:10.12688/f1000research.14776.2)
Supplement: Peer review history

# Peer review: Referee response for version 2

**DOI:** 10.5256/f1000research.16396.r34295

**Published:** 2018-06-11

**Authors:** John H. Morris

**Affiliations:** 1 Resource for Biocomputing, Visualization and Informatics, Department of Pharmaceutical Chemistry, University of California, San Francisco, San Francisco, CA, USA

I want to thank the authors for their changes in response to my review.  I believe the article now reads well and users will now be able to follow the specific steps and API calls in the workflow.

I have read this submission. I believe that I have an appropriate level of expertise to confirm that it is of an acceptable scientific standard.

# Peer review: Referee response for version 2

**DOI:** 10.5256/f1000research.16396.r34294

**Published:** 2018-05-23

**Authors:** Marina Piccirillo

**Affiliations:** 1 Lab-GTP, High Performance Computing and Networking Institute, National Research Council, Naples, Italy

I don't have any new comments.

I have read this submission. I believe that I have an appropriate level of expertise to confirm that it is of an acceptable scientific standard.

# Peer review: Referee response for version 1

**DOI:** 10.5256/f1000research.16077.r33666

**Published:** 2018-05-11

**Authors:** John H. Morris

**Affiliations:** 1 Resource for Biocomputing, Visualization and Informatics, Department of Pharmaceutical Chemistry, University of California, San Francisco, San Francisco, CA, USA

In this article, the authors have described their extensions to the popular ReactomeFIViz Cytoscape App to support automation via CyREST. They do an excellent job providing the motivation for their extension. The manuscript was clear and well-written, and I believe that the new REST API's will be quite useful.

 I do have a problem with the manuscript, however, in that in the Workflow section, they state:

 "The MAF was then loaded into Cytoscape via the CyREST API to construct a FI sub-network after choosing a sample cutoff to select genes forming a network composed of about 500 genes. The FI-network was then subject to network clustering analysis. Two sets of network modules from network clustering were compared to find modules shared and not shared between these two cancers. Pathway enrichment analysis was performed to collect pathways not shared between them."

 My problem with this is that they don't provide us with enough detail on the actual CyREST calls and parameters they used to allow us to understand how to reproduce it. Which part of their analysis was done in Python vs. which was done in Cytoscape through the GUI. It is true that all of my answers exist in the Jupyter notebook they provided, but that's a lot of work to figure out that they used the "buildFISubNetwork" method with the "MAF" format the fiVersion "2016".  My suggestion is to add at least enough information either in the figure or the text to help others understand the REST flow for their App without referring to the notebook.

I have read this submission. I believe that I have an appropriate level of expertise to confirm that it is of an acceptable scientific standard, however I have significant reservations, as outlined above.

# Response

**Published:** 2018-05-20

**Authors:** Guanming Wu

**Affiliations:** Oregon Health & Science University, USA

Dear Dr. Morris,

 Thanks a lot for reviewing our paper and your comment. In version 2 of our paper, we have added a new table listing detailed information about actions performed in the use case workflow (Table 2), and added new words in the main text (the second paragraph in the Workflow section) to help readers understand how to reproduce the workflow without going to the Jupyter notebook. We hope our revisions have addressed your concern.

 Best regards,

 Guanming Wu, Ph.D.

# Peer review: Referee response for version 1

**DOI:** 10.5256/f1000research.16077.r33745

**Published:** 2018-05-10

**Authors:** Marina Piccirillo

**Affiliations:** 1 Lab-GTP, High Performance Computing and Networking Institute, National Research Council, Naples, Italy

The authors describe the development of CyREST API for ReactomeFIViz, a Cytoscape app, which exposes some Reactome functions as REST APIs for external software components, to process pathway and network data in automatic and reproducible workflows built using almost any programming languages. The source code provided for ReactomeFiViz and for the Python use case notebook, make the use of CyREST API easier and simpler to understand.

 The manuscript is well-organized and the described app will be highly valuable for users working with big data related to enrichment analysis and visualization of networks.

 I have only some minor comments: 

1. In the Introduction section (pag.2): the sentence “These approaches project significant genes, proteins, metabolites, and other kinds of biological entities collected from pre-analysis onto pathways and networks, knowledge produced by many years’ experimental studies.”, is not quite understandable, I think it should be rephrased.
2. The Methods section (pag.2) is clearly explained and sufficient details are provided to allow replication of the method development and its use by others.
3. The section Operation (pag.2) I think is unnecessary, the authors can add it as the subsection of Methods because here they are explaining the methods available in the CyREST API developed.
4. In the Analysis results subsection (pag.3): I think it's possible to add a figure (such as a Venn Diagram) to better explain the results obtained.
5. In the same subsection (Analysis results: pag.3) more details should be provided about the results. (For example:
  -       in BRCA modules 13 and 17 are there overlapping genes? If yes, which are their names?
  -       What is the way, in which the enriched signaling pathways are involved in BRCA and/or OV carcinoma?)
6. More references to supporting literature must be provided in the manuscript in order to further substantiate the claims. 

I have read this submission. I believe that I have an appropriate level of expertise to confirm that it is of an acceptable scientific standard.

# Response

**Published:** 2018-05-20

**Authors:** Guanming Wu

**Affiliations:** Oregon Health & Science University, USA

Dear Dr. Piccirillo,

 Thanks a lot for reviewing our paper and your comments. We have made changes to address some of your comments. Please see details below:

 1). In the Introduction section (pag.2): the sentence “These approaches project significant genes, proteins, metabolites, and other kinds of biological entities collected from pre-analysis onto pathways and networks, knowledge produced by many years’ experimental studies.”, is not quite understandable, I think it should be rephrased.

***We have rephrased this sentence and hope the meaning is clear now.***

 2). The Methods section (pag.2) is clearly explained and sufficient details are provided to allow replication of the method development and its use by others.

***Thanks!***

 3). The section Operation (pag.2) I think is unnecessary, the authors can add it as the subsection of Methods because here they are explaining the methods available in the CyREST API developed.

***This section is suggested by the article template provided by the journal editor.***

 4). In the Analysis results subsection (pag.3): I think it's possible to add a figure (such as a Venn Diagram) to better explain the results obtained.

***Thanks for the suggestion. However, we found that it is difficult to show this type of overlapping result in a Venn diagram. We hope that our text description is clear.***

 5). In the same subsection (Analysis results: pag.3) more details should be provided about the results. (For example:

 -       in BRCA modules 13 and 17 are there overlapping genes? If yes, which are their names?

***We have added another sentence to address your question in the second paragraph of the Analysis Result section.***

 -       What is the way, in which the enriched signaling pathways are involved in BRCA and/or OV carcinoma?)

***This is a great question. However, this short software article is written to showcase how to use the new ReactomeFIViz CyREST API. We have refrained ourselves to discuss the biological implications of our analysis results. However, the exported pathway diagrams may answer questions like this, which will be addressed in our future projects.***

 6). More references to supporting literature must be provided in the manuscript in order to further substantiate the claims. 

***Thanks for the comment. Along with listed references, we have also linked to external resources using hyper-links in the main text. We feel that we have cited enough supporting references for this on-line short software article.***

 Best regards,

 Guanming Wu, Ph.D.
